# Hybridization in Parasites: Consequences for Adaptive Evolution, Pathogenesis, and Public Health in a Changing World

**DOI:** 10.1371/journal.ppat.1005098

**Published:** 2015-09-03

**Authors:** Kayla C. King, Rike B. Stelkens, Joanne P. Webster, Deborah F. Smith, Michael A. Brockhurst

**Affiliations:** 1 Department of Zoology, University of Oxford, Oxford, United Kingdom; 2 Max Planck Institute for Evolutionary Biology, Plön, Germany; 3 Department of Pathology and Pathogen Biology, Centre for Emerging, Endemic and Exotic Diseases (CEEED), Royal Veterinary College, University of London, London, United Kingdom; 4 Department of Biology, University of York, York, United Kingdom; The Fox Chase Cancer Center, UNITED STATES

Hybridization of parasites is an emerging public health concern at the interface of infectious disease biology and evolution. Increasing economic development, human migration, global trade, and climate change are all shifting the geographic distribution of existing human, livestock, companion animal, and wildlife parasites [1–9]. As a result, human populations encounter new infections more frequently, and coinfection by multiple parasites from different lineages or species within individual hosts occurs. Coinfection may have a large impact on the hosts and parasites involved, often as a result of synergistic or antagonistic interactions between parasites [10]. Indeed, mixed-species coinfections have been found to influence parasite establishment, growth, maturation, reproductive success, and/or drug efficacy [11–13]. However, coinfections can allow for heterospecific (between-species or between-lineage) mate pairings, resulting in parthenogenesis (asexual reproduction in which eggs occur without fertilization), introgression (the introduction of single genes or chromosomal regions from one species into that of another through repeated backcrossing), and whole-genome admixture through hybridization [14].

Recent molecular developments have revealed instances of fertile hybridization and introgression in plants [15], animals [16,17], and humans [18]. However, modern genetics and genomics have also uncovered the first confirmed cases of introgression within eukaryotic parasites [14,19]. Examples for such successful reticulate evolution in parasites include, but are not exclusive to, causative agents of important diseases initiated by fungi (*Cryptococcus* [20]), helminthic worms (*Schistosoma*, *Fasciola*, *Ascaris*, and *Trichinella* [21,22–24]), and protozoa (*Plasmodium*, *Leishmania*, *Toxoplasma*, and *Trypanosoma* [25,26–31]), as well as their vectors. These cases involve introgression between members of the neglected tropical diseases (NTDs) and/or neglected zoonotic diseases (NZDs)—highly debilitating diseases infecting more than a sixth of the world’s human population (and their livestock), with devastating consequences for individuals and communities. Such diseases are predominantly diseases of the world’s poorest communities, afflicting those people who are most at risk of contracting mixed parasitic infections (and hence also at risk for potential novel introgressed parasite infections) and at the same time least likely to get proper medical care once infected [32].

Evidence gathered to date, mainly from studies on nonparasitic animals and plants, suggests that hybridization can have a major evolutionary impact [33]. While hybridization can result in lower average fitness due to F1 sterility and inviability, caused by negative epistatic interactions [34–36] and the disruption of beneficial gene complexes [37,38], hybridization is at the same time a rich source of new genetic variation [39]. This can provide the raw material for natural selection to shape the evolution of ecologically relevant traits [33,40,41]. Thus, while a hybrid offspring, under certain conditions, may be less fit than its purebred offspring counterparts from either parental line, hybridization broadens the “working surface” for selection by producing a whole range of potentially adaptive phenotypes, ranging from one parent to the other and beyond. In nonparasitic taxa, hybridization has already been shown to promote geographic range expansions of populations (for example, in spiders [42]) as well as adaptation to new environments (Darwin finches [43]). There is also increasing evidence that hybridization can lead to the functional diversification of a group, as shown in *Helianthus* sunflowers [44], and even to speciation (reviewed in [40,45,46]). One key example of this is the case of “transgressive hybrids,” i.e., hybrids expressing extreme phenotypes that do not reside in either of the parental populations. These are “hopeful monsters” with respect to their evolutionary potential because they can diverge from their parents ecologically and may quickly become reproductively isolated from them [47–49]. In addition to broadening the selection surface, hybridization can also speed up the process of adaptation. After hybridization events, new alleles or allelic combinations that happen to be beneficial in the new environment are available immediately. This is in contrast to nonhybrid populations in which adaptation relies on alleles brought in via immigration or de novo mutation, both processes that require relatively longer periods of time [50,51]. As a consequence, populations with a hybrid origin may survive rapid environmental change better than their nonhybrid parents, as recently shown in yeast [52].

We predict that parasites are one of the major groups of organisms in which hybridization can have major impacts on the evolution and diversification of a group [14,19] and can lead to speciation [53], affecting key pathogenic traits and transmission. Hybridization in parasites may thus pose a serious challenge for the prevention, effective control, and therapy of disease [19]. While it has been suggested that hybridization and introgression between parasites can potentially drive the emergence and rapid evolution of novel zoonotic diseases [14], most studies to date are descriptive and do not consider the evolutionary consequences. Here, we review some of the most recent advances in the detection of hybridization in eukaryotic parasites (and their vectors) and discuss the significance of parasite hybridization for adaptive evolution and public health supported by relevant case studies in *Schistosoma* spp. (Box 1), *Leishmania* and *Trypanosoma* (Box 2), and the malaria vector *Anopheles* (Box 3). Given the role of infectious agents in our changing world, particularly in terms of emerging parasitic disease in response to anthropogenic change [32], it is time for a new and integrative perspective. Here, we argue for the integration of parasitology, disease biology, and evolutionary biology to understand the consequences of parasite hybridization to aid in the management and prevention of disease.

**Box 1. Case Example—Introgression in Helminths: Schistosomiasis**

*Schistosoma* spp. are the causative agents of schistosomiasis, a prevalent, chronic, and debilitating helminthic disease of humans and animals that occurs across much of the developing world. As early as 1948, there have been phenotypic reports of eggs indicative of potential *Schistosoma haematobium–S*. *mattheei* hybrids in Rhodesia/Zimbabwe [75], *S*. *bovis–S*. *curassoni* hybrids in West African ruminants, *S*. *haematobium–S*. *mattheei* hybrids in Southern African ruminants, and *S*. *haematobium–S*. *bovis* hybrids in humans from West Africa [76–78]. More recently, ITS1+2 and *cox1* barcoding studies of viable schistosome miracidial larvae hatched from the stool and urine of Senegalese school children confirmed bidirectional hybridization between human *S*. *haematocium* and livestock *S*. *bovis* [67], as well as for *S*. *haematobium* and *S*. *curassoni* [79]. Studies from infected snails in Kenya have observed hybrid cercariae between *S*. *mansoni* from humans and its sister species, *S*. *rodhaini*, from rodents [80]. These authors, using microsatellite markers (ribosomal DNA [rDNA] and mitochondrial DNA [mtDNA]), demonstrated that these hybrids produce viable offspring through first or successive generation backcrosses with *S*. *mansoni*. Unlike the *S*. *haematobium* and *S*. *bovis* or *S*. *currassoni* hybrids described above, the direction of introgression appeared highly asymmetric, causing unidirectional gene flow from the rodent *S*. *rodhaini* to the human *S*. *mansoni* [80]. Recent evidence from infected humans in Senegal has also revealed the potential for introgressions between the more phylogenetically distant pairings of the two major human schistosome species in Africa, *S*. *haematobium* and *S*. *mansoni*, a pairing previously thought to result in unviable eggs exclusively through parthogenesis [81]. These studies combined provide convincing evidence that schistosome species readily hybridize in nature, which may have major implications in light of the current global push for human disease control programmes to shift from controlling morbidity to halting transmission [32]. How such introgression may alter host range is perhaps the most pressing area for future research. Many schistosome species infecting livestock have a broader geographical range beyond Asia and Africa, with compatible snail intermediate hosts present. Novel zoonotic hybrids may therefore have the potential to be a global disease, particularly in our current climate of global warming and increased human and animal movement and transportation. This may be highlighted most clearly where novel introgressed hybrids between human *S*. *haematobium* and livestock *S*. *bovis* have recently been identified, with substantial ongoing transmission amongst both local residents and tourists, within Europe [82–84].

**Box 2. Case Example—Introgression in Protozoans: Leishmaniosis and Trypanosomiasis**

Introgressions can occur within the causative agents of protozoal diseases leishmaniasis and trypanosomiasis (Kinetoplastida: Trypanosomatidae) [85–88]. Approximately 30,000 people in 36 countries of sub-Saharan Africa suffer from human African trypanosomiasis (HAT), and Chagas disease has been classed as the most important vector-borne infection in Latin America, affecting an estimated 7–8 million humans, with around 21,000 deaths per year [89]. *Leishmania* parasites are another of the most important vector-borne pathogens in the developing world. Both *Leishmania* and *Trypanosoma* are also parasites with major zoonotic reservoirs. Two of the major lineages of *Trypanosoma cruzi* (discrete typing units [DTUs] III and IV) are now thought to have arisen by intraspecific hybridisation [87], despite their predominant mode of asexual reproduction, and introgression between subspecies has been associated with virulence [88]. Similarly, whilst asexual reproduction through clonal propagation has been proposed to be the major reproductive mechanism across the genus *Leishmania*, a hybridizing sexual cycle has been detected within its sand fly vector from across a range of geographical locations [90,91]. Whole-genome sequencing of *Leishmania* parasites isolated from sand flies from a Turkish endemic area indicated that variation in these parasites arose following a single cross between two phylogenetically distinct strains. Furthermore, whilst it appears that these populations do reproduce primarily clonally following this original hybridization event, subsequent recombination between the progeny does also occur [92]. The potentially large epidemiological consequences of such recombination events may be demonstrated by the observation that *Leishmania infantum/L*. *major* hybrids possess an enhanced host range, as hybrid offspring, and, unlike their parental single species, they are able to infect another vector, *Phlebotomus papatasi* [93].

**Box 3. Case Example—Introgression in the Parasite Vector *Anopheles*: Malaria**

*Anopheles* has become a model organism at the interface of speciation genomics and epidemiology [94], showcasing the potential perils of hybridization for public health but also the possible benefits of using controlled and induced hybridization as a means for disease control. *Anopheles* mosquitoes are vectors for malaria, which is caused by parasitic protozoans belonging to the genus *Plasmodium*. Malaria affects 200 million people a year worldwide, with an estimated 0.5–1 million deaths per year, mostly among young children in sub-Saharan Africa, where 90% of the world’s malaria deaths occur [95].

There are approximately 60 different species of *Anopheles* found worldwide [96], and genomic sequences of 16 *Anopheles* species (including vector and nonvector species) have recently become available [94,97]. These sequences have revealed fast and flexible evolutionary rates with respect to traits affecting their transmission potential, shown extensive introgression between *Anopheles* species, and suggested that enhanced vectorial capacity and adaptation to humans as primary hosts can result from interspecific genetic exchange. A multilocus single nucleotide polymorphism (SNP) genotyping panel is also at hand to detect F1 hybrids and backcrosses between the main vectors of malaria, *Anopheles gambiae* sensu stricto (S form) and *A*. *coluzzii* (M form) [98].

Control of *Anopheles* through insecticides, such as dichlorodiphenyltrichloroethane (DDT) and pyrethroids, has contributed to the prevention of malaria, but resistance to insecticides has recently emerged in *Anopheles* populations [99,100]. Recent studies have demonstrated rapid adaptive introgression of the insecticide resistance mutation *Vgsc-L1014F* from *A*. *gambiae* to *A*. *coluzzii*, in response to strong anthropogenic selection from increased insecticide use [101,102]. Others have found gene flow occurring at rates “far from inconsequential” between other species of *Anopheles* (e.g., between *A*. *gambiae and A*. *arabiensis* in Uganda [99,103] and *A*. *sinensis* and *A*. *kleini* in Korea [104]). Experimental interpopulation crosses of *A*. *gambiae*, monitored for traits determining their malaria transmission potential, scored higher for fecundity, body size, adult longevity, and average blood meal size, compared to both parental strains [105]. It is thus conceivable that the fitness-enhancing potential of hybridization may also apply to closely related interspecific hybrid crosses.

Hybridization between *Anopheles* species may have beneficial effects for disease control. Asymmetric introgression has recently been shown to transfer adaptive immunity and increased refractoriness to the *Plasmodium* pathogen from *A*. *coluzzii* to *A*. *gambiae* in Guinea [106]. Another opportunity for disease control is to modify the host preference of *A*. *gambiae* from humans to cattle by hybridizing it with *A*. *quadriannulatus*, a more zoophilic nonvector species, as a strategy to decrease its competence as malaria vector [107].

## Consequences of Hybridization in Parasites

Since multidrug resistance became a worldwide problem in pathogenic bacteria in the 1950s [54], we know that the exchange of genetic material via horizontal gene transfer among bacterial taxa has contributed to their evolution and pathogenesis [55,56]. Horizontal gene transfer can be advantageous and confer higher fitness, for instance, through the acquisition of antibiotic drug resistance [57] or through the spread of virulence factors; a well-studied example is the Shiga toxin genes exchanged between *Escherichia coli* and *Shigella* bacterial pathogens [58]. There are also several examples of recombinant human viruses that have exchanged genes with other strains with detrimental effects (Spanish flu, human rotavirus, and dengue fever; [59,60,61]). Conversely, despite their equally negative impact on host populations, not much is known about the hybridization of eukaryotic parasites, their frequency in the wild, or how hybridization may affect their spread and pathogenicity. Modern molecular techniques, however, can expose the signature of hybridization in the genome more rapidly and accurately, thereby increasing the number of recent reports of parasite hybridization.

**Fig 1:**
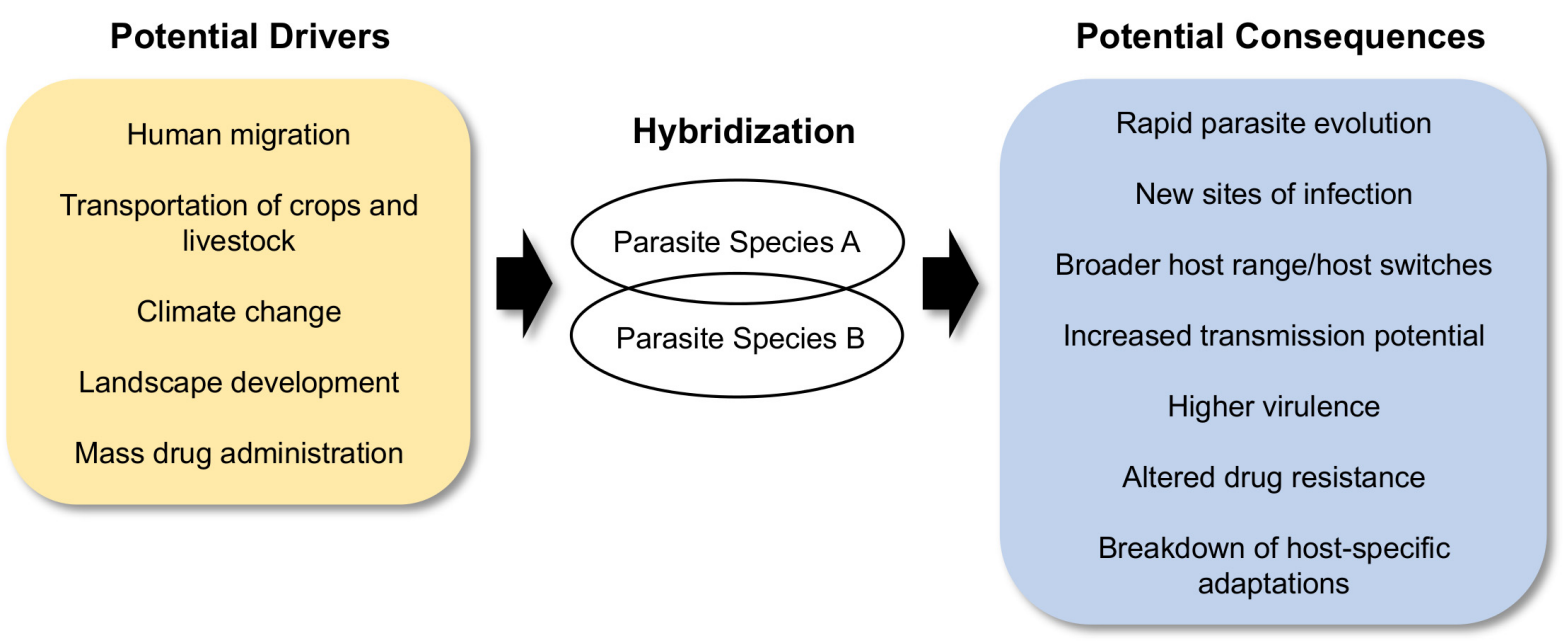
Schematic of the drivers and consequences of parasite hybridization.

We outline what we believe will be the most important and/or potentially dangerous effects of hybridization in eukaryotic parasites: (1) the generation of novel and extreme infection phenotypes, (2) an increase in host range, being just one component of (3) an increase in transmission potential, (4) an increase in parasite evolutionary potential with consequences for host–parasite coevolution, (5) the breakdown of host-specific adaptations, and also (6) an altered response to drug therapy (Fig 1).

1. As a result of new allelic combinations produced by hybridization, hybrid parasites may show enhanced phenotypic characteristics compared to the parents. Hybrids may be better at host exploitation, such that they may be more efficient at obtaining nutrition from the host, leading to higher fecundity or faster maturation time. Also, certain new hybrid parasite genotypes have been shown to be better at avoiding recognition and resistance from the host’s adaptive immune system, potentially leading to higher infectivity and unusual pathologies [29,62]. We thus predict that hybrid parasites are able to exploit novel resources and sites of infection within the host, which parental genotypes cannot utilize.
2. Hybrid parasites may be able to infect a greater range of host species [14,19,63]. While parental parasites are often restricted to one host species, hybrid parasites may be able to exploit both. Interspecific *Schistosoma* spp. hybrids, for instance, are able to infect both parental intermediate snail hosts (Box 1) [21]. In addition, just as some hybrids of nonparasitic taxa adapt to new ecological conditions and colonize novel environments that neither of the parents can survive [44,49], we would predict that hybrid parasites may be able to infect entirely new host species. As an indication that this indeed occurs in the wild, two plant pathogenic fungi (*Phytophthora* spp. infecting alder trees [64] and *Zymoseptoria pseudotritici* infecting a range of grasses [53]) and one amphibian fungal pathogen (a new lineage of *Batrachochytrium dendrobatidis* causing dramatic outbreaks of chytridiomycosis [65 *]*), which all emerged via hybridization, have been found to live on host species neither of their parents are found on.
3. With increasing land use and current rates of anthropogenic change across both the environment and agricultural/livestock practices, we predict that recombinant parasite genotypes will be generated at an increasing rate. Encounters between previously isolated parasite species become more frequent when geographic and ecological barriers that previously existed between parasite species are lost. Taking together the potential impact of hybrid superiority and host range expansions (described in 2) and an increase in anthropogenically mediated parasite dispersal, the transmission of disease may become considerably facilitated [66,67]. This may lead to epidemics, threaten global food security, and endanger natural animal and plant populations. For instance, the increasing creation and alteration of water bodies for agriculture can lead to areas of overlap and co-occurrence of the different intermediate snail host species of *Schistosoma* parasites [66,68]. This generates ample opportunity for coinfections of the definitive host by different *Schistosoma* species, which may then hybridize. In addition, humans and livestock are drawn to the same water resources, where they can both release parasites and become infected, thus creating a “hotbed” for disease transmission between human and livestock.
4. Evolutionary theory predicts that elevated virulence and genetic diversity, both potential outcomes of parasite hybridization, can increase the evolutionary potential of parasites and alter the host–parasite coevolutionary process [69]. For instance, for *Phytophthora*, hybridization has led to a range of new species with particularly aggressive pathogenicity [64], and hybrid lineages of the amphibian zoosporic fungus *Batrachochytrium*, which cause dramatic outbreaks of chytridiomycosis, have been found to be hypervirulent to many hosts [65]. In the context of coevolutionary races, higher virulence of hybrid parasites may impose stronger selection for host resistance.
5. Hybridization in parasites could, however, also be maladaptive for the parasites because of fitness-reducing genetic incompatibilities (negative epistasis) and the breakdown of host-specific adaptations, potentially leading to reduced infectivity/virulence. While such outbreeding depression is well known from free-living organisms, studies on the fitness of hybrid parasites are scarce. Outbreeding depression has been reported in a snail–trematode host–parasite system (*Potamopyrgus antipodarum–Microphallus* sp.), in which hybrid parasites suffer from reduced infectivity in both parental host populations [70,71]. In such cases, we predict that hybrid parasites may be less likely to evade host immunity or they may fail to effectively colonize the correct organ or body site. Through recombination and segregation, hybrids may also lose beneficial alleles that the parents had acquired previously, such as those conferring drug resistance (see below). Hybrid parasite inferiority can also lead to unusual pathogensis, which may hamper diagnosis, particularly when two very different disease phenotype parasites are introgressed (Box 1). As such, hybridization between divergent parasite populations may both promote and alter pathogenesis, which has important implications on disease prevalence, pathology, and treatment.
6. Hybridization may have a wide range of effects on drug efficacy in parasites. On the one hand, zoonotic hybrids may exhibit enhanced susceptibility to drugs if resistance alleles circulating in the human host parasite population are swamped by introgression of drug-susceptible alleles from the animal host parasite population. Furthermore, if hybridization between human and animal parasites increases the host range from human host specificity to that of a large animal host range reservoir, this could act against the establishment and spread of drug resistance [32]. Alternatively, hybridization could potentiate adaptive evolution within certain parasites in response to drug treatment (Box 1). One example for this is *Cryptococcus* serotype hybrids that are resistant to an antifungal drug usually used to treat infections with the individual parental serotypes [20]. More evidence for the adaptive introgression of drug resistance genes from nonsusceptible strains or species comes from *Haemonchus* nematodes that gained resistance against the broad-spectrum drug ivermectin through hybridization [72,73] and the introgression of anticoagulant rodent poison resistance by hybridization between Old World mice [74].

## Conclusions

Understanding and monitoring hybridization in animal and human parasites will be essential for optimizing and evaluating control strategies across potential hybrid zones. Relatively simple diagnostic procedures currently exist for detecting hybrids in some parasite groups (e.g., using a multilocus approach with mitochondrial cytochrome c oxidase subunit 1 (COX-1)/internal transcribed spacer (ITS) barcoding; [67]). However, the advancement of state-of-the-art genomic technologies will be helpful for more fine-scale detection of hybridization in parasites, to determine their evolutionary rates, and to identify patterns of cross transmission between host species (i.e., sources of transmission of zoonotic parasites to humans). Since hybrid parasites appear to be a particular problem in NTDs, there is a need to develop cheap, robust diagnostics appropriate to use in the field.

The circumstances producing increased opportunity for hybridization are the same ones that cause increased rates for disease distribution and transmission. Thus, the likelihood of parasite hybridization is increasing with the intensification of world trade of plants and animals, human migration, land use, and drug administration (Fig 1). Interspecific hybridization and introgression appear to be viable strategies for many parasites to maintain transmission, with potentially major impacts on the evolution of virulence, infection persistence, drug resistance, and host range, as well as on the clinical outcomes of disease. The combined effects of anthropogenic distribution and increased hybridization opportunities could have hazardous and catalytic effects on epidemiology, imposing further challenges and constraints for their effective control. More empirical work on the differential transmission, infectivity, drug efficacy, pathogenesis, and evolution of hybrid parasite populations is therefore urgently required to guide policies on their monitoring and management.

## References

[1] Semenza JC, Menne B (2009) Climate change and infectious diseases in Europe. Lancet Infectious Diseases 9: 365–375. 10.1016/S1473-3099(09)70104-5 19467476

[2] Nichols GL, Andersson Y, Lindgren E, Devaux I, Semenza JC (2014) European Monitoring Systems and Data for Assessing Environmental and Climate Impacts on Human Infectious Diseases. International Journal of Environmental Research and Public Health 11: 3894–3936. 10.3390/ijerph110403894 24722542 PMC4025019

[3] Martens P, Kovats RS, Nijhof S, de Vries P, Livermore MTJ, et al (1999) Climate change and future populations at risk of malaria. Global Environmental Change-Human and Policy Dimensions 9: S89–S107.

[4] Lafferty KD (2009) The ecology of climate change and infectious diseases. Ecology 90: 888–900. 19449681 10.1890/08-0079.1

[5] Brooks DR, Hoberg EP (2007) How will global climate change affect parasite-host assemblages? Trends in Parasitology 23: 571–574. 17962073 10.1016/j.pt.2007.08.016

[6] Patz JA, Graczyk TK, Geller N, Vittor AY (2000) Effects of environmental change on emerging parasitic diseases. International Journal for Parasitology 30: 1395–1405. 11113264 10.1016/s0020-7519(00)00141-7

[7] Hales S, de Wet N, Maindonald J, Woodward A (2002) Potential effect of population and climate changes on global distribution of dengue fever: an empirical model. Lancet 360: 830–834. 12243917 10.1016/S0140-6736(02)09964-6

[8] Astrom C, Rocklov J, Hales S, Beguin A, Louis V, et al (2012) Potential Distribution of Dengue Fever Under Scenarios of Climate Change and Economic Development. Ecohealth 9: 448–454. 10.1007/s10393-012-0808-0 23408100

[9] Moore S, Shrestha S, Tomlinson KW, Vuong H (2012) Predicting the effect of climate change on African trypanosomiasis: integrating epidemiology with parasite and vector biology. Journal of the Royal Society Interface 9: 817–830. 10.1098/rsif.2011.0654 PMC3306657 22072451

[10] Nowak MA, May RM (1994) Superinfection and the evolution of parasite virulence. Proceedings of the Royal Society of London B: Biological Sciences 255: 81–89. 10.1098/rspb.1994.0012 8153140

[11] Norton AJ, Webster JP, Kane R, Rollinson D (2008) Inter-specific parasite competition: mixed infections of *Schistosoma mansoni* and *S*. *rodhaini* in the definitive host. Parasitology 135: 1–12. 18215335 10.1017/S0031182007004118

[12] Webster JP, Gower CM, Norton AJ (2008) Application of evolutionary concepts to predicting and evaluating the impact of mass-chemotherapy schistosomiasis control programmes. Evolutionary Applications 1: 66–83. 10.1111/j.1752-4571.2007.00012.x 25567492 PMC3352399

[13] Koukounari A, Donnelly CA, Sacko M, Keita A, Landoure A, et al (2010) The impact of single versus mixed schistosome species infections on liver, spleen and bladder morbidity within Malian children pre- and post-praziquantel treatment. BMC Infectious Diseases 10: 227 10.1186/1471-2334-10-227 20670408 PMC2927598

[14] Detwiler JT, Criscione CD (2010) An infectious topic in reticulate evolution: introgression and hybridization in animal parasites. Genes 210: 102–123. 10.3390/genes1010102 PMC3960858 24710013

[15] Baack EJ, Rieseberg LH (2007) A genomic view of introgression and hybrid speciation. Current Opinion in Genetics & Development 17: 513–518. 17933508 10.1016/j.gde.2007.09.001 PMC2173880

[16] Arnold M (2004) Natural hybridization and the evolution of domesticated, pest and disease organisms. Molecular Ecology 13: 997–1007. 15078439 10.1111/j.1365-294X.2004.02145.x

[17] Mavarez J, Salazar CA, Bermingham E, Salcedo C, Jiggins CD, et al (2006) Speciation by hybridization in *Heliconius* butterflies. Nature 441: 868–871. 16778888 10.1038/nature04738

[18] Hawks J, Cochran G (2006) Dynamics of adaptive introgression from archaic to modern humans. Paleoanthropology 2006: 101–115.

[19] Arnold ML (2004) Natural hybridization and the evolution of domesticated, pest and disease organisms. Molecular Ecology 13: 997–1007. 15078439 10.1111/j.1365-294X.2004.02145.x

[20] Li W, Averette AF, Desnos-Ollivier M, Ni M, Dromer F, et al (2012) Genetic diversity and genomic plasticity of *Cyptococcus neoformans* AD hybrid strains. G3: Genes, Genomes, Genetics 2: 83–97 22384385 10.1534/g3.111.001255 PMC3276195

[21] Webster BL, Diaw OT, Seye MM, Webster JP, Rollinson D (2013) Introgressive hybridization of *Schistosoma haematobium* group species in Senegal: species barrier break down between ruminant and human schistosomes. PloS Neglected Tropical Diseases 7: e2110 10.1371/journal.pntd.0002110 23593513 PMC3617179

[22] Criscione CD, Anderson JD, Sudimack D, Peng W, Jha B, et al (2007) Disentangling hybridization and host colonization in parasitic roundworms of humans and pigs. Proceedings of the Royal Society B-Biological Sciences 274: 2669–2677. 10.1098/rspb.2007.0877 PMC2279219 17725977

[23] Le TH, De NV, Agatsuma T, Thi Nguyen TG, Nguyen QD, et al (2008) Human fascioliasis and the presence of hybrid/introgressed forms of *Fasciola hepatica* and *Fasciola gigantica* in Vietnam. International Journal for Parasitology 38: 725–730. 18031748 10.1016/j.ijpara.2007.10.003

[24] Dunams-Morel DB, Reichard MV, Torretti L, Zarlenga DS, Rosenthal BM (2012) Discernible but limited introgression has occurred where *Trichinella nativa* and the T6 genotype occur in sympatry. Infection, Genetics and Evolution 12: 530–538. 10.1016/j.meegid.2012.01.004 22266240

[25] Rogers MB, Downing T, Smith BA, Imamura H, Sanders M, et al (2014) Genomic confirmation of hybridisation and recent inbreeding in a vector-isolated *Leishmania* population PLoS Genetics 10: e1004092 10.1371/journal.pgen.1004092 24453988 PMC3894156

[26] Sturm NR, Vargas NS, Westenberger SJ, Zingales B, Campbell DA (2003) Evidence for multiple hybrid groups in *Trypanosoma cruzi* . International Journal for Parasitology 33: 269–279. 12670512 10.1016/s0020-7519(02)00264-3

[27] Machado CA, Ayala FJ (2001) Nucleotide sequences provide evidence of genetic exchange among distantly related lineages of *Trypanosoma cruzi* . Proceedings of the National Academy of Sciences of the United States of America 98: 7396–7401. 11416213 10.1073/pnas.121187198 PMC34680

[28] Gaunt MW, Yeo M, Frame IA, Stothard JR, Carrasco HJ, et al (2003) Mechanism of genetic exchange in American trypanosomes. Nature 421: 936–939. 12606999 10.1038/nature01438

[29] Grigg ME, Bonnefoy S, Hehl AB, Suzuki Y, Boothroyd JC (2001) Success and virulence in toxoplasma as the result of sexual recombination between two distinct ancestries. Science 294: 161–165. 11588262 10.1126/science.1061888

[30] Akopyants NS, Kimblin N, Secundino N, Patrick R, Peters N, et al (2009) Demonstration of genetic exchange during cyclical development of *Leishmania* in the sand fly vector. Science 324: 265–268. 10.1126/science.1169464 19359589 PMC2729066

[31] Ramiro RS, Khan SM, Franke-Faynard B, Janse CJ, Obbard DJ, et al (2015) Hybridization and pre-zygotic reproductive barriers in Plasmodium. Proceedings of the Royal Society Biological Sciences Series B 282. 10.1098/rspb.2014.3027 PMC4426616 25854886

[32] Webster JP, Molyneux D, Hotez PJ, Fenwick A (2014) The contribution of mass drug administration to global health—past, present and future. Philosophical Transactions of the Royal Society of London B Biological Sciences 369: 20130434 10.1098/rstb.2013.0434 24821920 PMC4024227

[33] Arnold ML (2006) Evolution through genetic exchange; Press OU, editor. Oxford.

[34] Dobzhansky T (1937) Genetic nature of species differences. American Naturalist 71: 404–420.

[35] Müller HJ (1942) Isolating mechanisms, evolution and temperature. Biological Symposium 6: 71–125.

[36] Coyne JA, Orr HA (2004) Speciation. Sunderland, MA: Sinauer Associates.

[37] Edmands S (2002) Does parental divergence predict reproductive compatibility? Trends in Ecology & Evolution 17: 520–527.

[38] Lynch M (1991) The genetic interpretation of inbreeding depression and outbreeding depression. Evolution 45: 622–629. 28568822 10.1111/j.1558-5646.1991.tb04333.x

[39] Barton NH (2001) The role of hybridization in evolution. Molecular Ecology 10: 551–568. 11298968 10.1046/j.1365-294x.2001.01216.x

[40] Abbott R, Albach D, Ansell S, Arntzen JW, Baird SJE, et al (2013) Hybridization and speciation. Journal of Evolutionary Biology 26: 229–246. 10.1111/j.1420-9101.2012.02599.x 23323997

[41] Seehausen O (2004) Hybridization and adaptive radiation. Trends in Ecology & Evolution 19: 198–207. 16701254 10.1016/j.tree.2004.01.003

[42] Krehenwinkel H, Tautz D (2013) Northern range expansion of European populations of the wasp spider *Argiope bruennichi* is associated with global warming correlated genetic admixture and population-specific temperature adaptations. Molecular Ecology 22: 2232–2248. 10.1111/mec.12223 23496675

[43] Grant BR, Grant PR (2008) Fission and fusion of Darwin's finches populations. Philosophical Transactions of the Royal Society B-Biological Sciences 363: 2821–2829. 10.1098/rstb.2008.0051 PMC2606742 18508750

[44] Rieseberg LH, Raymond O, Rosenthal DM, Lai Z, Livingstone K, et al (2003) Major ecological transitions in wild sunflowers facilitated by hybridization. Science 301: 1211–1216. 12907807 10.1126/science.1086949

[45] Mallet J (2007) Hybrid speciation. Nature 446: 279–283. 17361174 10.1038/nature05706

[46] Schumer M, Rosenthal GG, Andolfatto P (2014) How common is homoploid hybrid speciation? Evolution 68: 1553–1560. 10.1111/evo.12399 24620775

[47] Dittrich-Reed DR, Fitzpatrick BM (2012) Transgressive hybrids as hopeful monsters. Evolutionary Biology 40: 310–315. 23687396 10.1007/s11692-012-9209-0 PMC3655218

[48] Rieseberg LH, Archer MA, Wayne RK (1999) Transgressive segregation, adaptation and speciation. Heredity 83: 363–372. 10583537 10.1038/sj.hdy.6886170

[49] Stelkens RB, Brockhurst MA, Hurst GDD, Miller EL, Greig D (2014) The effect of hybrid transgression on environmental tolerance in experimental yeast crosses. Journal of Evolutionary Biology 27: 2507–2519. 10.1111/jeb.12494 25262771

[50] Barrett RDH, Schluter D (2008) Adaptation from standing genetic variation. Trends in Ecology & Evolution 23: 38–44. 18006185 10.1016/j.tree.2007.09.008

[51] Hedrick PW (2013) Adaptive introgression in animals: examples and comparison to new mutation and standing variation as sources of adaptive variation. Molecular Ecology 22: 4606–4618. 10.1111/mec.12415 23906376

[52] Stelkens RB, Brockhurst MA, Hurst GDD, Greig D (2014) Hybridization facilitates evolutionary rescue. Evolutionary Applications 7: 1209–1217. 10.1111/eva.12214 25558281 PMC4275092

[53] Stukenbrock EH, Christiansen FB, Hansen TT, Dutheil JY, Schierup MH (2012) Fusion of two divergent fungal individuals led to the recent emergence of a unique widespread pathogen species. Proceedings of the National Academy of Sciences of the United States of America 109: 10954–10959. 10.1073/pnas.1201403109 22711811 PMC3390827

[54] Davies J (1996) Origins and evolution of antibiotic resistance. Microbiologia 12: 9–16. 9019139

[55] Gyles C, Boerlin P (2014) Horizontally Transferred Genetic Elements and Their Role in Pathogenesis of Bacterial Disease. Veterinary Pathology Online 51: 328–340. 10.1177/0300985813511131 24318976

[56] Ochman H, Moran NA (2001) Genes lost and genes found: the molecular evolution of bacterial pathogenesis and symbiosis. Science 292: 1096–1098. 11352062 10.1126/science.1058543

[57] Ochman H, Lawrence JG, Groisman EA (2000) Lateral gene transfer and the nature of bacterial innovation. Nature 405: 299–304. 10830951 10.1038/35012500

[58] Strauch E, Lurz R, Beutin L (2001) Characterization of a Shiga Toxin-Encoding Temperate Bacteriophage of *Shigella sonnei* . Infection and Immunity 69: 7588–7595. 11705937 10.1128/IAI.69.12.7588-7595.2001 PMC98851

[59] Gibbs MJ, Armstrong JS, Gibbs AJ (2001) Recombination in the hemagglutinin gene of the 1918 "Spanish flu". Science 293: 1842–1845. 11546876 10.1126/science.1061662

[60] Laird AR, Ibarra V, Ruiz-Palacios G, Guerrero ML, Glass RI, et al (2003) Unexpected detection of animal VP7 genes among common rotavirus strains isolated from children in Mexico. Journal of Clinical Microbiology 41: 4400–4403. 12958276 10.1128/JCM.41.9.4400-4403.2003 PMC193830

[61] Worobey M, Rambaut A, Holmes EC (1999) Widespread intra-serotype recombination in natural populations of dengue virus. Proceedings of the National Academy of Sciences of the United States of America 96: 7352–7357. 10377418 10.1073/pnas.96.13.7352 PMC22089

[62] Schelkle B, Faria PJ, Johnson MB, van Oosterhout C, Cable J (2012) Mixed infections and hybridisation in monogenean parasites. Plos ONE 7: e39506 10.1371/journal.pone.0039506 22808040 PMC3394765

[63] Volf P, Benkova I, Myskova J, Sadlova J, Campino L, et al (2007) Increased transmission potential of *Leishmania major*/*Leishmania infantum* hybrids. International Journal for Parasitology 37: 589–593. 17376453 10.1016/j.ijpara.2007.02.002 PMC2839924

[64] Brasier CM, Cooke DEL, Duncan JM (1999) Origin of a new *Phytophthora* pathogen through interspecific hybridization (vol 96, pg 5878, 1999). Proceedings of the National Academy of Sciences of the United States of America 96: 13589–13589. 10.1073/pnas.96.10.5878 PMC21954 10318978

[65] Farrer RA, Weinert LA, Bielby J, Garner TWJ, Balloux F, et al (2011) Multiple emergences of genetically diverse amphibian-infecting chytrids include a globalized hypervirulent recombinant lineage. Proceedings of the National Academy of Sciences 108: 18732–18736. 10.1073/pnas.1111915108 PMC3219125 22065772

[66] Tchuente LAT, Southgate VR, Njiokou F, Njine T, Kouemeni LE, et al (1997) The evolution of schistosomiasis at Loum, Cameroon: replacement of *Schistosoma intercalatum* by *S*. *haematobium* through introgressive hybridization. Transactions of the Royal Society of Tropical Medicine and Hygiene 91: 664–665. 9509173 10.1016/s0035-9203(97)90513-7

[67] Huyse T, Webster BL, Geldof S, Stothard JR, Diaw OT, et al (2009) Birdirectional introgressive hybridization between a cattle and human schistosome species. PLoS Pathogens 5: e1000571 10.1371/journal.ppat.1000571 19730700 PMC2731855

[68] Webster BL, Tchuente LAT, Southgate VR (2007) A single-strand conformation polymorphism (SSCP) approach for investigating genetic interactions of *Schistosoma haematobium* and *Schistosoma guineensis* in Loum, Cameroon. Parasitology Research 100: 739–745. 17058111 10.1007/s00436-006-0310-0

[69] Lively CM (1999) Migration, virulence, and the geographic mosaic of adaptation by parasites. American Naturalist 153: S34–S47. 10.1086/303210 29578775

[70] Dybdahl MF, Jokela J, Delph LF, Koskella B, Lively CM (2008) Hybrid fitness in a locally adapted parasite. American Naturalist 172: 772–782. 10.1086/592866 18950274

[71] Lively CM, Dybdahl MF (2000) Parasite adaptation to locally common host genotypes. Nature 405: 679–681. 10864323 10.1038/35015069

[72] Redman E, Sargison N, Whitelaw F, Jackson F, Morrison A, et al (2012) Introgression of Ivermectin resistance genes into a susceptible *Haemonchus contortus* strain by multiple backcrossing. PloS Pathogens 8: e1002534 10.1371/journal.ppat.1002534 22359506 PMC3280990

[73] Chaudhry U, Redman E, Abbas M, Muthusamy R, Ashraf K, et al (2015) Genetic evidence for hybridisation between *Haemonchus contortus* and *Haemonchus placei* in natural field populations and its implications for interspecies transmission of anthelmintic resistance. International Journal for Parasitology 45: 149–159. 10.1016/j.ijpara.2014.09.002 25449043

[74] Song Y, Endepols S, Klemann N, Richter D, Matuschka F-R, et al (2011) Introgression of anticoagulant rodent poison resistance by hybridization between old world mice. Current Biology 21: 1296–1301. 10.1016/j.cub.2011.06.043 21782438 PMC3152605

[75] Alves W (1948) Transactions of the Royal Society of Tropical Medicine and Hygiene, 41: 430–439.

[76] Bremond P, Campagne G, Sellin B, Labbo R, Garba A, et al. (1996) Les schistosomes anthropophiles et zoophiles au Niger et leur impact sur la santé publique: détermination du risque réel de contamination et de pathogénicité pour les populations humaines. Rapport CERMES 6/96 document: 22 pages.

[77] Rollinson D, Southgate VR, Vercruysse J, Moore PJ (1990) Observations on natural and experimental interactions between *Schistosoma bovis* and *Schistosoma curassoni* from West-Africa. Acta Tropica 47: 101–114. 1969699 10.1016/0001-706x(90)90072-8

[78] Taylor MG (1970) Hybridisation experiments on five species of african schistosomes. Journal of Helminthology 17: 253–314. 10.1017/s0022149x00021969 5505355

[79] Webster BL, Diaw OT, Seye MM, Webster JP, Rollinson D (2013) Introgressive hybridization of *Schistosoma haematobium* group species in Senegal: species barrier break down between ruminant and human schistosomes. PLoS Neglected Tropical Diseases 7: e2110 10.1371/journal.pntd.0002110 23593513 PMC3617179

[80] Steinauer ML, Hanelt B, Mwangi IN, Maina GM, Agola LE, et al (2008) Introgressive hybridization of human and rodent schistosome parasites in western Kenya. Molecular Ecology 17: 5062–5064. 10.1111/j.1365-294X.2008.03957.x 18992007

[81] Huyse T, Van den Broeck F, Hellemans B, Volckaert FAM, Polman K (2013) Hybridisation between the two major African schistosome species of humans. International Journal of Parasitology 43: 687–689. 10.1016/j.ijpara.2013.04.001 23643461

[82] Berry A, Moné H, Iriart X, Mouahid G, Abbo O, et al (2014) *Schistosomiasis haematobium*, Corsica, France [letter]. Emerging Infectious Diseases 20: 1595–1597. 10.3201/eid2009.140928 25153697 PMC4178424

[83] ECDC (2014) Rapid risk assessment: Local transmission of *Schistosoma haematobium* in Corsica, France– 16 May 2014. Stockholm.

[84] Boissier J, Moné H, Mitta G, Bargues MD, Molyneux DH, et al (2015) Schistosomiasis reaches Europe. Lancet Infectious Diseases 15: 757–758. 10.1016/S1473-3099(15)00084-5 26122434

[85] Messenger LA, Garcia L, Vanhove M, Huaranca C, Bustamante M, et al (2015) Ecological host fitting of *Trypanosoma cruz*i TcI in Bolivia: mosaic population structure, hybridization and a role for humans in Andean parasite dispersal. Molecular Ecology 24: 2406–2422. 10.1111/mec.13186 25847086 PMC4737126

[86] Messenger LA, Llewellyn MS, Bhattacharyya T, Franzén O, Lewis MD, et al (2012) Multiple mitochondrial introgression events and heteroplasmy in *Trypanosoma cruzi* revealed by Maxicircle MLST and Next Generation Sequencing. PLoS Neglected Tropical Diseases 6: e1584 10.1371/journal.pntd.0001584 22506081 PMC3323513

[87] Miles MA, Llewellyn MS, Lewis MD, al. e (2009) The molecular epidemiology and phylogeography of Trypanosoma cruzi and parallel research on Leishmania: looking back and to the future. Parasitology 136: 1509–1528. 10.1017/S0031182009990977 19691868

[88] Goodhead I, Capewell P, Bailey JW, Beament T, Chance M, et al (2013) Whole-genome sequencing of *Trypanosoma brucei* reveals introgression between subspecies that is associated with virulence. mBio 4: e00197–13. 10.1128/mBio.00197-13 23963174 PMC3747575

[89] WHO (2014) Global Burden of Disease Estimates for 2000–2012.

[90] Kelly JM, Law JM, Chapman CJ, Van Eys GJ, Evans DA (1991) Evidence of genetic recombination in Leishmania. Molecular and Biochemical Parasitology 46: 253–263. 1656255 10.1016/0166-6851(91)90049-c

[91] Inbar E, Akopyants NS, Charmoy M, Romano A, Lawyer P, et al (2013) The Mating Competence of Geographically Diverse Leishmania major Strains in Their Natural and Unnatural Sand Fly Vectors. PLoS Genetics 9: e1003672 10.1371/journal.pgen.1003672 23935521 PMC3723561

[92] Rogers MB, Downing T, Smith BA, Imamura H, Sanders M, et al (2014) Genomic confirmation of hybridisation and recent Inbreeding in a vector-isolated *Leishmania* population. PLoS Genetics 10: e1004092 10.1371/journal.pgen.1004092 24453988 PMC3894156

[93] Volf P, Benkova I, Myskova J, Sadlova J, Campino L, et al (2007) Increased transmission potential of *Leishmania major/Leishmania infantum* hybrids. Intenational Journal for Parasitology 37: 589–593. 10.1016/j.ijpara.2007.02.002 PMC2839924 17376453

[94] Fontaine MC, Pease JB, Steele A, Waterhouse RM, Neafsey DE, et al (2015) Extensive introgression in a malaria vector species complex revealed by phylogenomics. Science 347: 1258524 10.1126/science.1258524 25431491 PMC4380269

[95] World Health Organization. Malaria 2015 [updated 2015; cited 2015 23 June]. http://www.who.int/malaria/en/.

[96] Manguin S, Carnivale P, Mouchet J (2008) Biodiversity of Malaria in the World; John Libbey Eurotext, editor. Paris, France

[97] Neafsey DE, Waterhouse RM, Abai MR, Aganezov SS, Alekseyev MA, et al (2015) Highly evolvable malaria vectors: The genomes of 16 *Anopheles* mosquitoes. Science 347: 1258522 10.1126/science.1258522 25554792 PMC4380271

[98] Lee Y, Marsden CD, Nieman C, Lanzaro GC (2014) A new multiplex SNP genotyping assay for detecting hybridization and introgression between the M and S molecular forms of *Anopheles gambiae* . Molecular Ecology Resources 14: 297–305. 10.1111/1755-0998.12181 24119184 PMC3947471

[99] Mawejje HD, Wilding CS, Rippon EJ, Hughes A, Weetman D, et al (2013) Insecticide resistance monitoring of field-collected *Anopheles gambiae s*.*l*. populations from Jinja, eastern Uganda, identifies high levels of pyrethroid resistance. Medical and Veterinary Entomology 27: 276–283. 10.1111/j.1365-2915.2012.01055.x 23046446 PMC3543752

[100] Wondji CS, Coleman M, Kleinschmidt I, Mzilahowa T, Irving H, et al (2012) Impact of pyrethroid resistance on operational malaria control in Malawi. Proceedings of the National Academy of Sciences 109: 19063–19070. 10.1073/pnas.1217229109 PMC3511128 23118337

[101] Clarkson CS, Weetman D, Essandoh J, Yawson AE, Maslen G, et al (2014) Adaptive introgression between *Anopheles* sibling species eliminates a major genomic island but not reproductive isolation. Nat Commun 5: 4248 10.1038/ncomms5248 24963649 PMC4086683

[102] Norris LC, Main BJ, Lee Y, Collier TC, Fofana A, et al (2015) Adaptive introgression in an African malaria mosquito coincident with the increased usage of insecticide-treated bed nets. Proceedings of the National Academy of Sciences 112: 815–820. 10.1073/pnas.1418892112 PMC4311837 25561525

[103] Weetman D, Steen K, Rippon E, Mawejje H, Donnelly M, et al (2014) Contemporary gene flow between wild *An*. *gambiae s*.*s*. and *An*. *arabiensis* . Parasites & Vectors 7: 345. 25060488 10.1186/1756-3305-7-345 PMC4124135

[104] Choochote W, Min G-S, Intapan P, Tantrawatpan C, Saeung A, et al (2014) Evidence to support natural hybridization between *Anopheles sinensis* and *Anopheles kleini* (Diptera: Culicidae): possibly a significant mechanism for gene introgression in sympatric populations. Parasites & Vectors 7: 36. 24443885 10.1186/1756-3305-7-36 PMC3899613

[105] Menge D, Guda T, Zhong D, Pai A, Zhou G, et al (2005) Fitness consequences of *Anopheles gambiae* population hybridization. Malaria Journal 4: 44 16174295 10.1186/1475-2875-4-44 PMC1242248

[106] Mancini E, Spinaci MI, Gordicho V, Caputo B, Pombi M, et al (2015) Adaptive Potential of Hybridization among Malaria Vectors: Introgression at the Immune Locus *TEP1* between *Anopheles coluzzii* and *A*. *gambiae* in ‘Far-West’ Africa. PLoS ONE 10: e0127804 10.1371/journal.pone.0127804 26047479 PMC4457524

[107] Pates HV, Curtis CF, Takken W (2014) Hybridization studies to modify the host preference of *Anopheles gambiae* . Medical and Veterinary Entomology 28: 68–74. 10.1111/mve.12070 25171608

